# Pharmaceutical policy and innovation for rare diseases: A narrative review

**DOI:** 10.12688/f1000research.130809.1

**Published:** 2023-02-24

**Authors:** Adrián Alonso Ruiz, Kaitlin Large, Suerie Moon, Marcela Vieira

**Affiliations:** 1 Global Health Centre, Graduate Institute of International and Development Studies, Geneva, 1211, Switzerland

**Keywords:** health policy, rare diseases, pharmaceutical innovation, pharmaceutical policy, innovation policy, access to medicines

## Abstract

This article aims to synthesize the existing literature on the implementation of public policies to incentivize the development of treatments for rare diseases, (which are diseases with very low prevalence and therefore with low commercial interest) otherwise known as orphan drugs. The implementation of these incentives in the 1980s in the United States (US), later in Japan, and in the European Union (EU) seems to be related to a substantial increase in treatments for these diseases, and has influenced the way the pharmaceutical research & development (R&D) system operates beyond this area. In spite of this success, the academic literature also highlights the negative implications that these public policies have on affordability and access to orphan drugs, as well as on the prioritization of certain disease rare areas over others.

The synthesis focuses mostly on the United States’ Orphan Drug Act (ODA) as a model for subsequent policies in other regions and countries. It starts with a historical overview of the creation of the term “rare diseases”, continues with a summary of the evidence available on the US ODA’s positive and negative impacts, and provides a summary of the different proposals to reform these incentives in light of the negative outcomes described. Finally, it describes some key aspects of the Japanese and European policies, as well as some of the challenges captured in the literature related to their impact in Low- and Middle-Income Countries (LMICs).

## Introduction

This literature synthesis aims to map, summarize, and disseminate the available evidence of policies incentivizing orphan drug R&D. In addition, the purpose of the synthesis is to examine the extent, range, breadth, depth, and nature of these policies.

## Methods

We followed the framework for scoping studies proposed by Arksey and O’Malley (2005), consisting of five stages: identifying the research question(s), identifying relevant studies, study selection, data charting and summarizing, and reporting the results ( Arksey & O’Malley, 2005).

Our two research questions were the following:

• What is the impact of R&D incentives on the development of therapies for rare diseases?
• What is the evidence regarding the impact of these incentives on affordability and global access?

The research questions were open-ended enough to capture evidence from different countries and regions, and understand the context in which these incentives were developed, the actors involved, and the positive and negative impacts of these public policies, therefore achieving a good balance between depth and breadth of scope.

In order to answer these questions, different keywords related to the following concepts were used and combined to construct the search syntax: Research and Development (R&D) incentives (search terms used: “research and development incentiv*” OR “Research and Development polic*” OR “drug development” OR “drug innovation”), therapies for Rare Diseases (search terms used: “Orphan Drug Act” OR “Orphan Drug Legislation” OR incentiv* OR “Orphan drug” OR “Rare disease therap*” OR “Rare disease*” OR “orphan drug designation”), affordability, and accessibility (search terms used: Affordab* OR pric* AND “global*” “access*”).

The search of relevant literature was done in PubMed by using different permutations of the selected search terms to build an initial database of articles. The language of the articles selected was English, and no gray literature was included (see Limitations section). We included all articles regardless of the year of publication. Additionally, all relevant articles identified were mapped using Litmaps, a tool that provides a visualization of how publications are connected by their reference lists and suggests relevant articles based on these connections.

**Figure 1.**
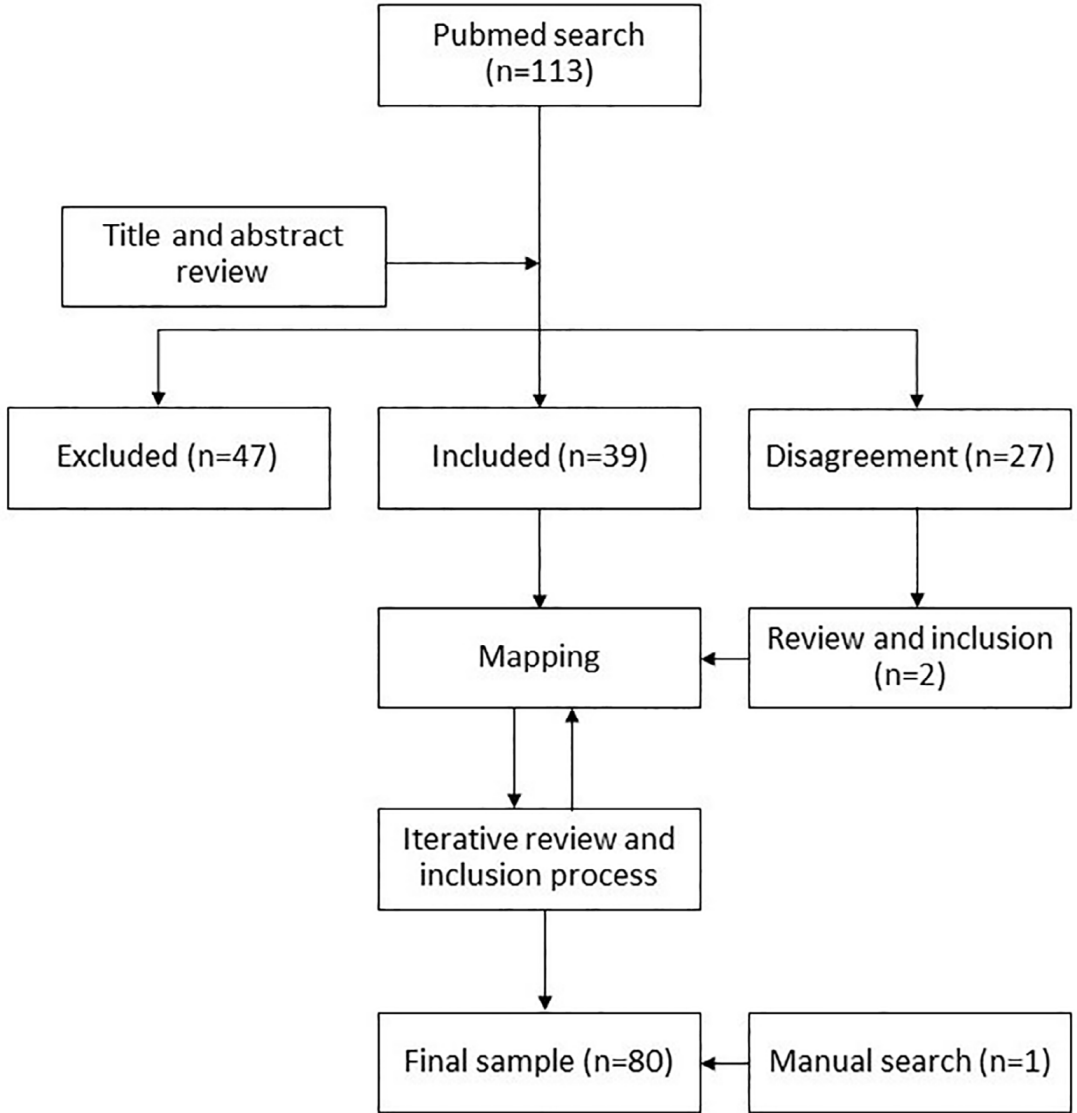
Review flowchart.

The initial database from PubMed contained 113 articles. Two researchers (AR and KL) separately reviewed the titles and abstracts of this initial corpus of articles to select those that could address the research questions, while excluding publications that focused solely on problems related to downstream access issues (see Limitations). The two reviewers had a 76% overlap in their decisions, excluding 47 articles and including 39 that became the initial body of literature. The remaining 27 articles where there was a discrepancy between the two reviewers were subject to a second review by AR at a later stage, which resulted in the inclusion of two more articles ( Figure 1).

**Figure 2.**
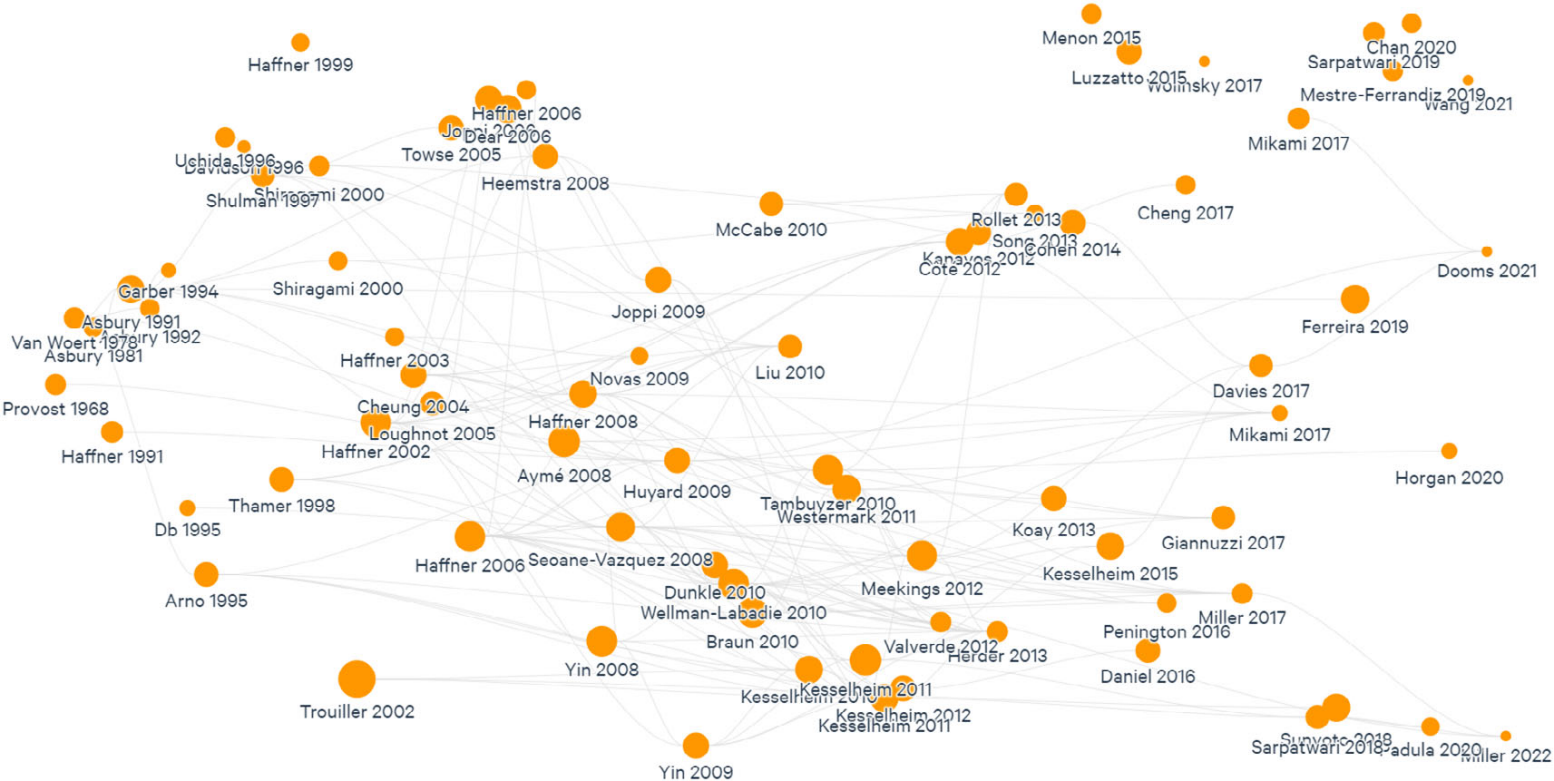
Mapping of studies included in this review.

As one researcher read the 39 articles, some cross-cutting themes started to be identified through an inductive process and charted in a summary document. The themes are presented in this review as the headings in the Results section. This led to an iterative process consisting of reading and analyzing the studies to identify themes, visualize the reference list on Litmaps, and include relevant studies to gain depth into a specific theme. This process was done until saturation was reached. This ensured a more consistent and comprehensive list of studies for the themes identified ( Figure 2). Finally, information on the burden of rare diseases globally was included by manually searching for relevant literature.

## Results

### 1. Defining rare diseases

Concerns about the lack of economic viability of pharmaceutical R&D in certain areas started emerging in the late 1960s in the US. According to several authors, the implementation of the Kefauver-Harris Drug Amendments in 1962 – which increased regulatory standards for drug safety and efficacy – led to an increase in development and regulatory costs for the industry ( Arno *et al*., 1995; Asbury, 1981, 1992; Cheung *et al*., 2004; Haffner *et al*., 2002; Huyard, 2009; Loughnot, 2005; Mikami, 2017; Van Woert, 1978).

As a byproduct of this regulatory change, the pharmaceutical industry shifted the priorities of its business model, pivoting around three axes: (a) the anticipated time and resources needed to meet safety and efficacy requirements relative to market size; (b) patentability of the candidates; and (c) legal liability risks after commercialization ( Asbury, 1981).

There were two main consequences related to this shift: first, the creation of a group of treatments known to be effective but not compliant with the new regulation, named “homeless” or “orphan” treatments, as their development was not commercially attractive for the industry ( Cheung *et al*., 2004; Huyard, 2009; Loughnot, 2005; Mikami, 2017; Provost, 1968; Van Woert, 1978). Second, the abandonment of the development of “service drugs” (treatments with public health relevance, but with small profitability prospects) as a regularized practice in the industry. This affected drugs treating diseases with low prevalence ( Asbury, 1981, 1991, 1992; Huyard, 2009; Mikami, 2017; Provost, 1968; Van Woert, 1978), but also those treating larger target populations ( Arno *et al*., 1995; Asbury, 1991; Haffner, 1991; Huyard, 2009), such as vaccines and “drugs for developing nations” ( Asbury, 1981; Huyard, 2009).

Since there was no pre-existing medical category for these diseases, which had in common the lack of commercial interest and industrial neglect, policymakers had to define the set of diseases that would be targeted in the development of public policies to incentivize drug development. The implementation of the Orphan Drug Act (ODA) in the US was the first formal categorization of these diseases (which are now referred to as rare diseases) and influenced the development of similar legislation in other countries. The ODA used prevalence as the main criteria to assess whether one disease is considered rare or not, therefore excluding other diseases that had been neglected for other reasons ( Herder, 2013; Huyard, 2009).

Huyard argues that “rare disease” is not a term derived from a “refinement in medical knowledge” and “remains meaningless for physicians”. Rather, the creation and use of the term is purposely blurry to foster cooperation between different stakeholders ( Huyard, 2009). The creation of this category has highlighted the situation of some patients and facilitated the adoption of public policies to address their needs at the national level (or regional level in the case of the EU).

However, it has also created difficulties to measure the burden of rare diseases globally, as the prevalence thresholds change from one country to another (a disease considered rare in the US might not be rare in Japan or the EU and vice versa), or as the categorization of diseases change (a disease that was not rare in the past may have been divided into different sub-diseases that qualify as rare) ( Ferreira, 2019). Ferreira (2019) estimates there are between 5,000-8,000 rare diseases, and establishes a cumulative prevalence of 6.2% over the global population (acknowledging several limitations to the methodology).

### 2. The US Orphan Drug Act

The US Orphan Drug Act was approved in 1983 to stimulate the development of drugs and biologicals for US patients with rare diseases and conditions. The Act defined rare diseases as “diseases or circumstances which occur so infrequently in the USA, that there is no reasonable expectation that the cost of developing and making available in the USA a drug for such disease or condition will be recovered from sales in the USA for such drugs” ( Clissold, 1995).

The 1983 Act included a set of provisions and incentives to offset and reduce the risk of investment and increase revenues for products that were unprofitable and non-patentable ( Asbury, 1992; Clissold, 1995). The following summary of provisions and incentives comes from Arno *et al*., 1995; Asbury, 1991, 1992; Haffner, 1991, 1999; Loughnot, 2005; Thamer *et al*., 1998:

• Protocol assistance from the US Food and Drug Administration (FDA) during the development before or after a drug candidate has been designated an Orphan Drug.
• Seven-year market exclusivity upon commercialization that was initially only applicable to unpatented products, but later in a 1985 amendment extended to include all orphan drugs.
• Tax credits on clinical trial expenses that cover 50% of all costs. The other 50% of the costs were considered deductible, which overall reduced tax liability by approximately 70%. In 2017, the tax credit was reduced from 50% to 25% ( Padula *et al*., 2020).
• Open protocols that permitted researchers to include patients during clinical trials to provide access to orphan drugs for treatment purposes.
• Research grants to cover costs of preclinical and clinical testing of drugs, medical devices, and medical foods.
• Creation of administrative bodies such as the Orphan Products Board in the Department of Health and Human Services, the Office of Orphan Products Development (OOPD) at the FDA (that manages the ODA incentives and designates orphan drugs), and the National Commission on Orphan Diseases, that supports, coordinates, and stimulates orphan drug development.

Additionally, in 1997 in a separate piece of legislation, the FDA Modernization Act exempted all orphan drugs from application fees ( Haffner, 1999, 2003).

In order to obtain an Orphan Drug Designation (ODD), the 1983 ODA required applicants to justify lack of economic viability for the indication that the product was targeting ( Asbury, 1992; Haffner, 1991). However, in 1984 the Act was amended to include a new designation mechanism for drugs that treated diseases with a prevalence below 200,000 patients ( Arno *et al*., 1995; Clissold, 1995; Haffner, 1991; Herder, 2013; Huyard, 2009). Some authors claim that this change was sought by the industry in order to avoid scrutiny over companies’ financial data, with sponsors not including the figures in the dossier ( Arno *et al*., 1995; Haffner, 1991; Herder, 2013; Mikami, 2017). According to Mikami, the 200,000 limit was set by policymakers in order to capture certain diseases such as Tourette Syndrome, multiple sclerosis, and narcolepsy ( Dunkle *et al*., 2010), after the OOPD proposed a lower prevalence (100,000 patients) ( Mikami, 2017).

In 1985, another amendment expanded market exclusivity to patented orphan products ( Clissold, 1995). This amendment, together with the definition of rare disease based on prevalence, de facto assumed that any drug targeting a rare disease is not profitable ( Herder, 2013).

2.1. Impact of the ODA on the pharmaceutical innovation system

Several authors portrayed the ODA as a successful policy to foster innovation in the pharmaceutical sector, but also raised concerns over several negative consequences of the ODA shortly after its implementation. This section attempts to collate both the positive and negative impacts of the ODA that appear in the literature.

Most authors use the substantial increase in orphan products developed as a measure of the success of the ODA. These results started emerging shortly after the bill was passed, and many other authors have supported this idea of success since then ( Braun *et al*., 2010; Cheung *et al*., 2004; Côté & Keating, 2012; Haffner, 2003, 2006; Haffner *et al*., 2002; Haffner & Maher, 2006; Shulman & Manocchia, 1997; Thamer *et al*., 1998; Wellman-Labadie & Zhou, 2010). However, most of these authors do not aim to study the aggregate impact of the ODA on increasing innovative activity ( Yin, 2008), but rather to provide a description of the increase of orphan drug approvals since the ODA’s implementation.

Some authors point out that in addition to the likely positive effect of the ODA, other “confounding factors” such as other laws (Waxman-Hatch Act, Prescription Drug User Fee Act, Small Business Innovation Development Act), governmental bodies (e.g., Orphan Drugs Board, NIH ORD) or the role of patient groups could have contributed as well to the overall increase of orphan drugs ( Kesselheim, 2011; Seoane-Vazquez *et al*., 2008). Zhang and Wang (2021) exemplified this complex network of actors and incentives that contribute to the development of orphan drugs, through the story of the development of Epogen ( Zhang & Wang, 2021).

For instance, the public and academic sector contribution have also been deemed essential in the basic science and preclinical stages. Kesselheim *et al*. (2015) investigated the development stories of several breakthrough therapies (including several orphan drugs), finding that “many of the key insights behind these transformative products emerged in publicly funded basic research in university settings, and were then further developed through collaboration between public and private entities” ( Kesselheim *et al*., 2015). According to Haffner (2003), through the FDA’s grant program 33 supported orphan products had received marketing authorization by January 2003 ( Haffner, 2003).

The chronological description provided by Wellman-Labadie and Zhou in 2010, showed that the increase in orphan drug designations and approvals was not constant but three-phased, hypothesizing that phases of stagnation or decrease in ODDs followed financial or economic instability. In addition, proposed amendments to the ODA in the 1990s that could have modified some of the incentives included in the bill, seemed to coincide with a decrease in the number of designations and approvals ( Wellman-Labadie & Zhou, 2010). The relation between the stability of the law and its capacity to attract companies and investors has been mentioned in other authors’ works as well ( Clissold, 1995; Towse & Kettler, 2005).

The impact of market exclusivity had not been evaluated until the late 2000s, despite the fact that many authors considered it the most powerful incentive ( Arno *et al*., 1995; Asbury, 1991, 1992; Clissold, 1995; Haffner, 1991). Seoane and collaborators (2008) challenged the idea, providing an evaluation of the market exclusivity provision, and suggesting that the Orphan Drug Act exclusivity provided a “modest” increase of between 0.8 and 0.9 years of market exclusivity to orphan new molecular entities (NMEs), concluding that market exclusivity was longer than patent protection in only one in seven drugs included in the study. However, although the authors reported that the maximum effective patent life and market exclusivity life was significantly lower for orphan drugs compared to non-orphan drugs, orphan NMEs had significantly less generic competition compared to non-orphan NMEs ( Seoane-Vazquez *et al*., 2008).

Sarpatwari and collaborators (2018) showed a similar trend in an analysis that included orphan drugs approved between 1985 and 2014. According to the authors, exclusivity protection granted under ODA lasted longer than patent protection for only one-third of the 160 drugs included in the study, and accounted for 17% of their total market exclusivity. Although the analysis did not include biologic drugs (which is recognized as a significant limitation), the authors suggested that the increase in OD small molecules approved had little relation with market exclusivity, and highlighted other factors such as high prices, lower development costs, and other incentives included in the ODA as potential contributors to the increase of orphan drug approvals ( Sarpatwari *et al*., 2018).

Padula and collaborators (2020) provide one of the most recent analyses of the market exclusivity incentive. On average, orphan drugs had 1.47 orphan approvals (as ODDs are granted per indication, one product can obtain several ODDs) and an extended market exclusivity of 1.6 years (considering that NCEs receive five years of market exclusivity). However, 25% of all drugs approved had two or more orphan drug designations, which increased its market exclusivity period between 4.7 years after the initial approval with two designations, and 13.4 years for drugs with five designations. Seven drugs obtained market exclusivity for two decades or more by accumulating orphan drug designations ( Padula *et al*., 2020).

Beyond the length of exclusivity protection, Miller (2017) showed that on average, companies’ stock prices increased 3.36% after the announcement of an orphan designation. Her study concludes that ODDs have a positive value to attract investors and signal future profitability, especially in the case of smaller companies ( Miller, 2017).

With respect to other incentives included in the Act, other authors mention the relevance of the FDA’s Orphan Products Grants Program, implemented under the ODA. The program has had an increasing budget (from $500,000 USD in 1983 to $30 million USD in 2012 ( Arno *et al*., 1995; Valverde *et al*., 2012) that covered up to $150,000 USD per year for Phase 1 trials, and up to $300,000 USD for Phase 2/3 in 2000 ( Haffner *et al*., 2002). Daniel and collaborators (2016) report that a company could receive up to $500,000 USD per year over four years as part of the grant program ( Daniel *et al*., 2016). Other programs, such as the National Institutes of Health’s (NIH) National Center for Advancing Translational Sciences, funded preclinical and clinical development of drugs for rare diseases ( Valverde *et al*., 2012).

Yin (2008) evaluated the impact of the ODA’s tax incentives in increasing R&D flow (measured as new clinical drug trials for a given disease in a given year). The author estimated an average increase of 69% in annual flow of new clinical trials for a list of “long-established” rare diseases, specifically in those with higher prevalence. In diseases with lower prevalence, the ODA led to an increase in the number of drugs available shortly after the ODA’s approval, but lower R&D activity throughout his study. The author highlights the role of tax credits to boost R&D capacity in rare diseases with higher market potential, but suggests their limited impact in developing drugs with limited demand or reduced profitability ( Yin, 2008).

In addition to the increase in orphan drug development and approval, the literature shows some unexpected effects that are partly attributed to the ODA, on the pharmaceutical R&D system. Some authors hypothesize that the contribution of the ODA combined with the scientific developments in biomedicine during the 1970s and 1980s, led to the emergence of the biopharmaceutical sector in the US and the relevance of small and medium enterprises in the US’ pharmaceutical innovation system. One reason for this is the growth of the share of biologic products developed by small and medium enterprises that obtained ODD ( Haffner *et al*., 2002; Haffner & Maher, 2006; Herder, 2013; Mikami, 2017; Seoane-Vazquez *et al*., 2008; Shulman & Manocchia, 1997; Valverde *et al*., 2012). Additionally, the willingness to develop the biopharmaceutical sector seems to be one of the reasons why similar legislations were approved in different countries and regions ( Davidson, 1996; Huyard, 2009; Mikami, 2017; Uchida, 1996).

Furthermore, these changes seem to be coupled with a change in the distribution of tasks in the R&D process. Wellman-Labadie and Zhou (2010) showed that biotechnology companies sponsored 73% of all ODDs between 1983 and 2009, but approvals were split evenly between them and pharmaceutical companies. ^1^ In addition, the top 10 pharmaceutical companies accounted for 26% of all orphan drug approvals, whereas the top 10 biopharmaceutical companies accounted for 12% of all approvals. These authors, as well as others, conclude that whereas smaller, usually biotechnology-focused R&D companies focus more on earlier stages of R&D, larger pharmaceutical companies focused more on in-licensing and marketing of these products ( Heemstra *et al*., 2008; Valverde *et al*., 2012; Wellman-Labadie & Zhou, 2010).

Finally, the introduction of the ODA also enabled a broader change in the pharmaceutical R&D business model. This change allowed companies to shift from a model based on mass production and sales of drugs for general care, to targeted drugs for rare diseases (or subdivisions of non-rare diseases) that are highly profitable ( Côté & Keating, 2012; Herder, 2013), as discussed in the following section.

2.2. Negative consequences of the ODA

Although many authors consider the ODA and similar pieces of legislation as successful policies for promoting drug R&D, negative consequences of the Act were raised shortly after its implementation. Five main issues arose before the 2000s and are here summarized from Arno *et al*., 1995; Asbury, 1991, 1992; Clissold, 1995; Garber, 1994; Haffner, 1991; Shulman & Manocchia, 1997.

First, the market exclusivity clause has been portrayed as the major contributor to the success of the ODA, but also as a major driver of unaffordable prices, as the monopolies allow companies to set high prices without competition.

Second, determining whether two orphan drugs intended for the same indication are the “same” drug became a matter of litigation, as many orphan drugs were biologic products (as compared to traditional chemically synthesized drugs) and there was a lack of guidance on how or whether it was possible to define similarity between two biologic products. This issue has been quite relevant as having at least two similar products approved for the same indication could reduce prices through competition, and increase access by having more products on the market. The FDA resolved in 1992 that similar drugs can obtain ODD only if the second product proves to be clinically superior to the marketed drug, therefore equating different with clinically superior, and granting an even broader protection to orphan drug sponsors ( Herder, 2013; Loughnot, 2005), given that as reported by Kesselheim (2011), the designation of a clinically superior biologic never happened in practice ( Kesselheim, 2011).

Third, the decision to provide orphan drug status to products based on disease prevalence led to concerns after the 1983 amendment, after some governmental bodies expressed apprehension about the impact of the new designation mechanism on drug prices, turning orphan drugs into “profitable drugs at some point” ( Clissold, 1995). This was due to the fact that on the one hand, drugs that treat diseases whose prevalence increases over the 200,000 limit could become highly profitable and still benefit from the ODA incentive package, as was the case of treatments for certain infectious diseases such as HIV.

On the other hand, the fragmentation of non-orphan diseases into smaller sub-diseases —a practice denominated ‘salami slicing’ ( Haffner & Maher, 2006; Loughnot, 2005)— to obtain several ODDs was observed in treatments for HIV and HIV-associated diseases in 1995, with treatments such as pentamidine or dapsone that received one designation to treat and another to prevent Pneumocystis pneumonia, as two separate indications. In this sense, Yin demonstrated that companies are incentivized to subdivide only those diseases with prevalence slightly higher than 200,000 patients ( Yin, 2009). This issue was also acknowledged by the FDA in 2011, claiming that the ODA precipitated “the creation of subsets of non-rare diseases or conditions that are artificially narrow” ( Herder, 2013). Miller and collaborators (2022) provide examples of orphan drugs that have received more than 10 ODDs from a single sponsor, three monoclonal antibodies (brentuximab, nivolumab, and pembrolizumab) and one small molecule, ibrutinib, that target different types of cancer ( Miller *et al*., 2022).

Finally, although the number of ODDs and orphan drugs approved had increased overall, not all disease categories increased at the same pace. Oncology represented 24% and 31% of all ODDs, and 23% and 25.5% of all orphan drug approvals in 1995 and 2007 respectively ( Seoane-Vazquez *et al*., 2008; Shulman & Manocchia, 1997). Wellman-Labadie and Zhou (2010) reported similar results in a retrospective study between 1983 to 2009 (32% of all designations and 26% of all approvals) ( Wellman-Labadie & Zhou, 2010). Miller (2017) showed that the increase in companies’ stock prices when obtaining ODD for an oncological product was larger and followed an upward trend compared to non-oncological products, showing that although investors seem to value ODDs, more value is attributed to oncological ODDs ( Miller, 2017).

These four issues kept emerging after the turn of the millennium ( Braun *et al*., 2010; Cheung *et al*., 2004; Côté & Keating, 2012; Daniel *et al*., 2016; Davies *et al*., 2017; Haffner *et al*., 2008; Kesselheim, 2010; Loughnot, 2005; McCabe *et al*., 2010; Wellman-Labadie & Zhou, 2010; Yin, 2009), and as evidence kept growing and new regions and countries implemented similar pieces of legislation, new issues emerged.

Although it had been reflected in the literature before ( Shulman & Manocchia, 1997), issues related to regulatory timelines, clinical trial design and patient enrollment, and quality of regulatory dossiers started being present in the literature in the 2000s. Given the small and geographically dispersed population, orphan drug developers had problems with ensuring adequate, well-controlled trials ( Dear *et al*., 2006; Haffner, 2003, 2006; Joppi *et al*., 2006). Joppi and collaborators (2006 and 2009) provided an overview of the issue in the European region, concluding that the quality of the regulatory dossiers for orphan drugs in the region was “poor” ( Joppi *et al*., 2006, 2009). Kesselheim *et al*., (2011) describe a similar picture in the US, showing statistical significance in the differences between orphan and non-orphan pre-approval trial design and characteristics, with smaller sample sizes, less randomization and blinding, and use of surrogate end points ( Kesselheim *et al*., 2011). Additionally, Yin (2009) highlighted the inefficiencies of the ODA incentives, showing that 10% of the clinical trials of treatments for these diseases would have been conducted in the absence of the ODA, but still received public subsidies ( Yin, 2009).

Joppi *et al.* (2006) attribute the “poor” quality of clinical trials to the lack of tax exemptions in Europe to improve the quality of the trials, ultimately linking them to a slower approval rate in Europe compared to the US ( Joppi *et al*., 2006). Wellman-Labadie and Zhou (2010) report that although the speed of development from ODD to commercialization is similar in the US and EU, the number of orphan drugs approved/designated is much lower. The authors point out that this may be a multicausal effect, but also included the lack of tax exemptions as one of the potential contributing factors ( Wellman-Labadie & Zhou, 2010).

On the contrary, Heemstra and collaborators (2008) modeled the predictors of orphan drug approval in the European region, and although they found that having orphan drugs already approved was the best predictor of success (companies with orphan drugs on the market had 17-fold more chances of having another orphan drug approved), they did not find any correlation between tax exemptions and market approvals ( Heemstra *et al*., 2008).

Regarding regulatory timelines, some studies have shown a slower approval timeline when considering clinical and approval phases together for orphan drugs ( Shulman & Manocchia, 1997). However, the approval phase seems to be faster for orphan drugs compared to non-orphan, given the use of accelerated regulatory pathways (e.g., Fast Track, Priority Review procedures) ( Seoane-Vazquez *et al*., 2008; Shulman & Manocchia, 1997).

Regarding orphan drugs and profitability, 18 out of the 43 blockbusters (drugs with global annual sales over $1 billion USD) with orphan drug designation analyzed in 2010, were approved only as orphan drugs. Eleven of these 18 reached blockbuster status within the market exclusivity period and 14 had at least two ODDs ( Wellman-Labadie & Zhou, 2010). Sarpatwari and collaborators mention the differences between orphan and non-orphan list prices (annual average per patient) in the US in 2014: $118,820 USD for Orphan and $23,331 USD for non-orphan ( Sarpatwari *et al*., 2018).

In their evaluation of the economic impact of the accumulation of the ODA’s market exclusivity provisions, Padula and collaborators (2020) estimated a $591.1 billion USD expenditure on all orphan drugs and orphan indications if prices were to be maintained after the first seven years of exclusivity ( Padula *et al*., 2020). Finally, Mestre-Ferrándiz *et al* (2019) showed a general increase in total pharmaceutical expenditure on Orphan Medicinal Products (OMP) in Europe from 2000 to 2017, with an increase 4% higher in new OMPs compared to non-OMPs over the entire analysis period ( Mestre-Ferrandiz *et al*., 2019).

All these different issues are intertwined, and during the 2010s, these practices were common across orphan drug developers, and the impact of this business model on access and affordability became a recurrent theme in the literature. As Côté and Keating (2012) describe, “orphan drug policies have the paradoxical effect of creating new orphan patients” as “actual legislations trigger a three-step strategy: 1) apply for orphan designation, obtain substantial economic benefits during the development, approval, and marketing phases, and demand a high price because of the low prevalence of the initial target population; 2) after approval, convince doctors to use the drug in their practice; and 3) expand sales by obtaining new therapeutic indications, orphan or otherwise, while maintaining the initial price”. According to the authors, this strategy is explained by the combined effect of the regulatory incentives, the excessive stratification of indications, pricing policies based on willingness to pay from purchasers, and the increase in off-label use for these treatments ( Côté & Keating, 2012).

**Figure 3.**
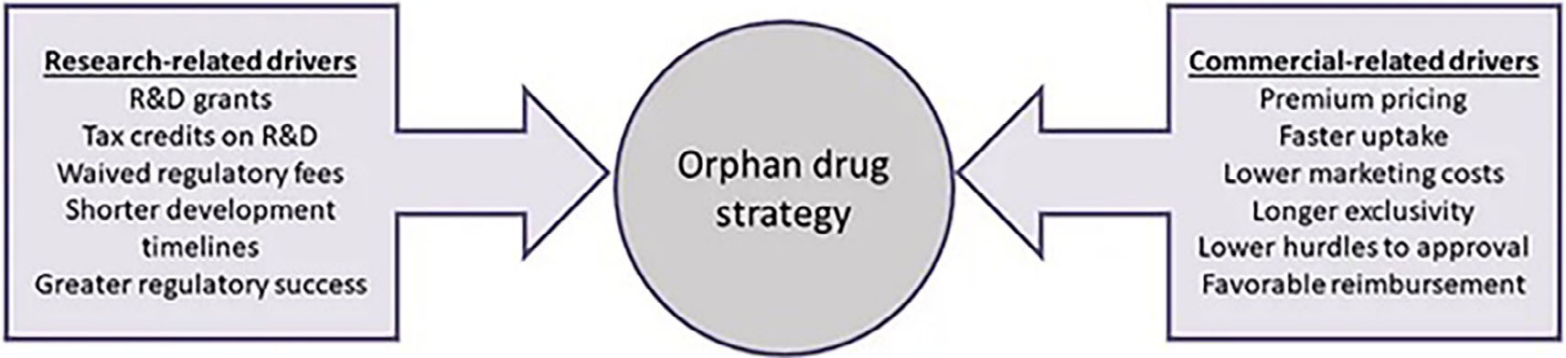
This figure has been adapted from Meekings, K. N., Williams, C. S. M., & Arrowsmith, J. E. (2012). Orphan drug development: An economically viable strategy for biopharma R&D. Drug Discovery Today, 17(13), 660–664 with permission from Drug Discovery Today (Elsevier).

These findings were partly corroborated by Kesselheim and collaborators (2012), as they found that the off-label use of orphan drugs greatly surpassed the approved use in two of the three drugs included in their study ( Kesselheim *et al*., 2012). Yin assessed this issue in a larger sample, demonstrating that diseases with prevalence slightly over 200,000 were more likely to be subdivided, and that treatments for these diseases had higher off-label sales ( Yin, 2009). From an industry perspective, Meekings and collaborators validate Côté and Keating’s framework, arguing that Orphan drug development can have greater profitability potential considering the financial incentives, smaller clinical trial sizes and times, and higher rates of regulatory success ( Figure 3) ( Meekings *et al*., 2012).

Daniel and collaborators (2016) and Penington (2016) support these ideas by providing examples of different orphan drugs priced at very high levels, and raising the issue of orphan drug prices increasing at a faster pace than non-orphan drugs ( Penington *et al*., 2016). As pointed out by other authors previously, obtaining multiple ODDs for the same drug, extending off-label use for increasingly compartmentalized diseases, and market exclusivity seem to be some of the causal factors ( Daniel *et al*., 2016).

In response to Côté and Keating, Kanavos and Nicod (2012) suggest that the focus in oncology might also be due to an increase in incidence or a consequence of the role of public sector investments in early stage and basic research of cancer drugs ( Kanavos & Nicod, 2012), an issue first raised in the late 1980s ( Mikami, 2017). In addition, the authors also consider that the lack of benchmarks to assess whether prices and/or company revenues are high or low impede the ability to know if prices meet the expectations of value generated to society ( Kanavos & Nicod, 2012). Similarly, and from an industry perspective, Rollet and collaborators (2013) and Tambuyzer (2010) acknowledge high prices of some orphan drugs, but describe the pricing of pharmaceuticals not as a function of the cost of development, but as a set of “multi-factorial” issues that include the value of therapeutics to payers, the return on the investment for shareholders, and the reinvestment in R&D for the company ( Rollet *et al*., 2013; Tambuyzer, 2010). ^2^

2.3. Potential reforms to the ODA

Given some of the issues associated with the ODA, there have been several attempts and proposals to reform it. Different amendments were proposed in the US Congress to mitigate some of the negative effects of the ODA and are often reflected in the literature. These attempted to create different “triggers” to terminate market exclusivity, such as obtaining over $200 million USD in revenues from sales of one product, or exceeding the prevalence threshold of 200,000 patients for a particular indication. Others included a “windfall tax” on all revenues obtained by a company from selling the orphan drug when these surpass a certain level (e.g., cost of development) ( Loughnot, 2005; Sarpatwari & Kesselheim, 2019). In 1990, US Congress passed one amendment to allow shared exclusivity to products developed simultaneously, and with a trigger to withdraw market exclusivity if disease prevalence increased over 200,000 patients. However, President Bush vetoed the amendment ( Mikami, 2017).

Daniel and collaborators (2016) and Loughnot (2005) proposed changes to the FDA’s procedures to grant orphan drug designation, including requiring sponsors to specify whether the disease that the drug is intended to treat is a subset of a broader indication. Although the FDA added in 2013 a section in the OD application requiring sponsors to report whether the indication was a subset of a larger disease ( Daniel *et al*., 2016), the present review did not find any evaluations of this change. The authors also suggest normative changes in how diseases are categorized (specifically in oncology), by focusing on the genetic cause rather than organ or origin of the cancer. They also suggest increased regulatory scrutiny, stratified incentives based on disease prevalence, decreased exclusivity periods, increased price transparency, and other incentives based on profit and disease prevalence thresholds ( Daniel *et al*., 2016; Loughnot, 2005).

Davies and collaborators propose that not-for-profit and socially motivated organizations from both the public and private sectors should play a greater role in the development and marketing of orphan drugs, especially in the repurposing field. The authors recognize that a lack of funding and knowledge of the regulatory and IP system can be significant barriers for these types of organizations, but they are not “insurmountable” ( Davies *et al*., 2017).

Herder suggests that integrating “ethical, value-type considerations and […] public participation into innovation design processes will precipitate better healthcare options and help legitimize, in a democratic sense, inexorably difficult resource allocation decisions in healthcare” ( Herder, 2013). More specifically, the author proposes a more preeminent role for the public sector through three different approaches:

• A modified version of the Health Impact Fund that would decouple medicine prices from the financial rewards for developers, which would be based on the health impact of having essential medicines accessible to those who need them.
• Grant-and-Access Pathway: proposed by Valverde *et al.* (2012), is based on the substitution of the tax credit by a robust grant program that included price caps. This mechanism would allow other actors such as universities, smaller firms, and start-ups to fully develop their products and enter the market. These actors usually don’t benefit from the tax credit for clinical trial expenses because they lack the revenue stream from other products to get to that stage, often relying on partnering, licensing, merging, or being acquired by a larger company ( Valverde *et al*., 2012).
• Publicly Funded Trials: through the creation of a network of companies that operated under long-term contracts of eight to 12 years with the government, or directly run by governmental or non-profit institutions, this model aims to increase clinical trials openness and transparency.

Hendrickx and collaborators (2021) propose the use of pharmaceutical compounding not as an alternative, but as a parallel way of developing and providing access to drugs and substances that are known to have activity for certain rare diseases. The authors view pharmaceutical compounding as a practice of self-governing (within the health system) a pharmaceutical commons, as the knowledge and goods to produce these drugs are widely accessible compared to the more privatized industrial system that results in the “enclosure of common therapies” ( Hendrickx *et al*., 2021).

### 3. Role of patients in orphan drug R&D

Several authors highlighted the vital role that patient groups play in both policymaking and the R&D process for orphan drugs. While the roles of research institutions, pharmaceutical companies, and public institutions are more apparent, patient groups worked to raise awareness about rare diseases and bring them to the forefront of the health policy agenda in the US. Specifically, they have accomplished these feats through the power of community organizing and advocating for policies that promoted positive change in the rare disease drug development space ( Arno *et al*., 1995; Asbury, 1992; Aymé *et al*., 2008; Dunkle *et al*., 2010; Mikami, 2017; Mikami & Sturdy, 2017; Novas, 2009). As Novas (2009) argues, “patients’ organizations have managed to integrate themselves into the relays of power through which matters of health are thought about and acted upon. Through their formation into coalitions, patients’ organizations have been able to assume a number of important functions in relation to the government of health” ( Novas, 2009). In Europe, patient organizations were actively involved in the development of the European Orphan Medicinal Products (OMP) legislation, which actually includes three representatives of patient organizations as members of the Committee for Orphan Medicinal Products (COMP), the body responsible for conferring Orphan Medicinal Product designation ( Mikami & Sturdy, 2017).

Koay and Sharp (2013) have studied the involvement of patient organizations in the R&D process for rare genetic diseases. The authors found that their involvement contributes to improving several aspects of the R&D process, from study design and data collection to dissemination of findings and assessment of research impact and outcomes ( Koay & Sharp, 2013).

Some of the articles reviewed provided examples of areas in the R&D process in which patient organizations have been involved, ranging anywhere from founding a start-up company or initiating clinical trials to move potential drug candidates to the market, to supporting, funding, and sharing registries and natural history databases to help academics or companies develop therapies ( Mikami & Sturdy, 2017).

Menon and collaborators (2015) give more detail on the contributions that patient organizations can make to improve information systems. Through sharing natural history registries, suggesting endpoints in trial design, reporting on outcomes and subjective data (patient-reported outcomes), etc., patients can help reduce uncertainties in the decision-making process for orphan drugs ( Menon *et al*., 2015).

Another role developed by patient associations has been to fund and facilitate the development of many orphan drugs ( Aymé *et al*., 2008; Koay & Sharp, 2013). The Cystic Fibrosis Foundation ( Côté & Keating, 2012; Wolinsky, 2017) is one example of a “venture philanthropic” organization, created by patients and patients’ relatives, that invests in companies that develop orphan drugs but also facilitates the coordination of clinical trials or the dissemination of results. As part of these investments, several orphan drugs have been commercialized for the treatment of Cystic Fibrosis and its complications. However, ethical issues arose as the organization obtained billions of dollars through the sale of royalties of these highly priced drugs ( Cohen & Raftery, 2014; Luzzatto *et al*., 2015). Additional concerns were raised by Mikami and Sturdy (2017) especially when considering the potential conflicts of interest arising in the collaboration between pharmaceutical companies and patient organizations ( Mikami & Sturdy, 2017).

Overall, there seems to be consensus in the role of patient groups to add value and reduce uncertainties and costs in the orphan drug development process. This consensus seems to be coupled with an equal concern about the risk of conflicts of interest in the interphase between these groups and the pharmaceutical industry.

### 4. Orphan drug legislation in other countries/regions

According to Chan and collaborators (2020), there are at least 162 countries/areas with some type of orphan drug policy in place. In their analysis, the authors grouped the different aspects of the policies together in six major themes: orphan drug designation (89.1% of countries studied), marketing authorization (84.8%), safety and efficacy requirements (47.8%), incentives that encourage R&D (47.8%), incentives to encourage market availability (46.7%), and price regulation (22.8%).

Almost all the countries with “incentives that encourage R&D” are High-Income Countries, with two Upper-Middle-Income Countries (Bulgaria, Romania) and two Lower-Middle Income Countries (Philippines, Vietnam). The incentives found in the literature as part of “the incentives that encourage R&D” theme were “patent protection/marketing exclusivity/monopolization”, “funding for research/development/clinical trials”, “scientific advice/consultation”, “protocol assistance”, and “national plan or strategy”.

Finally, the authors highlighted the importance of having a comprehensive orphan drug policy in place to not only overcome the barriers that these diseases face, but also to ensure that the unintended perverse effects are under control. For example, this could be the reason why many HICs face pricing issues, as this area is often neglected in these countries, according to the authors. On the other hand, it could explain why R&D in these diseases is less present in non-HICs, or why these treatments are overall less available, as these areas are often less developed ( Chan *et al*., 2020).

The following section will briefly discuss the Japanese and European Orphan Drug legislations, as these appeared most often in the literature. Although they are quite similar to the US ODA, they do offer some distinct features.

4.1. The Japanese Orphan Drug Act

The Japanese Orphan Drug Act came into effect in 1993, and is the second piece of legislation that specifically addressed the issue of orphan drug R&D. Closely related to the ODA, the Japanese legislation grants orphan drug status to drugs that meet the following criteria ( Thamer *et al*., 1998; Uchida, 1996): The drug must treat a disease with a prevalence below 50,000 patients in Japan and must be in need, meaning that it must be of therapeutic superiority or address a lack of therapeutic alternatives. Finally, the drug must have a high probability of successful development, and its efficacy needs to be supported by enough evidence in order to obtain orphan drug status.

After a drug is granted Orphan Status, the sponsor is entitled to the following incentives ( Herder, 2013; Thamer *et al*., 1998; Uchida, 1996):

• Access to public grants to cover R&D expenses that can cover up to 50% of all development costs.
• Tax exemptions for clinical development expenses that can cover 12% of the costs (excluding those covered by governmental grants), and a 14% reduction in corporate tax ( Song *et al*., 2013).
• Regulatory guidance and advice to facilitate the preclinical and clinical development of orphan drugs, and user fee waivers.
• Priority review and fast track procedures for new drug approval applications of orphan drugs.
• A 10-year market exclusivity period. However, in Japan the market exclusivity period for all drugs is six years before a reexamination period ( Shiragami & Nakai, 2000a).

One feature that is different from the US ODA is the inclusion of a “windfall tax”, mandating companies to pay a 1% sales tax on orphan drugs that have annual profits exceeding 100 million Japanese yen until the subsidies provided by the government are paid back ( Cheung *et al*., 2004; Daniel *et al*., 2016; Thamer *et al*., 1998; Uchida, 1996).

In 2000, Shiragami and Nakai replicated in Japan the analysis done by Shulman and collaborators (1997) in the US, analyzing the results of orphan drug development between 1983 and 1995. With fewer drugs designated in Japan compared to the US, treatments for HIV/AIDS and related diseases received the most designations, followed by genetic (including cancer) and metabolic diseases. By 1995, only 28.6% of the drugs studied were developed in Japan, and the majority of the companies filing for ODD were large companies ( Shiragami & Nakai, 2000a). Murakami and Narukawa (2016) reach similar conclusions, as the top 10 pharmaceutical companies by revenue in 2013 held 34.9% of all designations (compared to 9.9% in the EU or 15.4% in the US). Regarding therapeutic classification, antineoplastic and immunomodulating agents represented 31% of all designations, anti-infectives for systemic use represented 16.5%, and orphan drugs targeting diseases of the nervous system represented 10.7% ( Murakami & Narukawa, 2016).

The same authors measured in 2000 the impact of the incentive package for orphan drugs in Japan, concluding that orphan drugs represented an increasing amount of the total drugs approved: from 4.2% on average between 1980 to 1985 when the regulation was issued, to 19.0% between 1993 to 1999. The median value for regulatory review period was also significantly reduced from 26 months to 15.5 months in the same periods of time ( Shiragami & Nakai, 2000b). These results led the authors to conclude that the “support system” designed for the development of orphan drugs in Japan had been successful.

4.2. European incentives for orphan drug development

Introduced in the year 1999, the European Union’s Regulation No 141/2000 on Orphan Medicinal Products (OMPs) set very similar incentives as the US ODA ( Cheung *et al*., 2004; Dear *et al*., 2006). It sets four criteria to designate an orphan drug ( Committee for Orphan Medicinal Products and the European Medicines *et al*., 2011):

• Prevalence criterion: when the drug is intended to treat, diagnose, or prevent “a life-threatening or chronically debilitating” disease that affects not more than five per 10,000 people in the community when the application is made.
• Insufficient return on investment criterion: when the commercialization of the drug is unlikely to generate sufficient return to justify the necessary investment. As of 2011, the meaning of “sufficiently profitable” has not been defined and no other articles in the synthesis have provided more information on this matter.
• No satisfactory method criterion: when there is no other method of diagnosis, prevention, or treatment for the condition.
• Significant benefit criterion: when there is a diagnostic, preventive, or treating method, but the new product would bring significant benefit to those affected by the condition.

The regulation provides the sponsor with a 10-year market exclusivity period after market approval, which can be increased by two more years if the drug is for a pediatric orphan medicine. In contrast to the ODA, market exclusivity can be reduced to six years if the drug is considered sufficiently profitable. Market exclusivity can also be derogated because of the lack of supply of the product, and if a new product is proven to be “clinically superior”.

On the regulatory side, the Regulation provides protocol assistance and fee waivers (100% waiver for protocol assistance and 50% for all regulatory fees), which is highlighted as a strong incentive for small- and medium-sized enterprises. Protocol assistance can relate to the support for regulatory requirements such as product quality, preclinical studies, clinical trial design, etc. On the regulatory aspect, the EU regulation allows orphan products to be approved through the centralized procedure, which gives access to the 29 EU member states, Norway, and Iceland.

Finally, the EU has supported rare disease research through grant programs similar to the ones the US ODA offers, and provides fee waivers for pre- and post-authorization inspections and annual fees during the first year after market authorization ( Giannuzzi *et al*., 2017). However, one area where there is a discrepancy between the US ODA and the EU Regulation is with tax exemptions, as these cannot be imposed by the European Union and therefore are not included in the Orphan Regulation. The European Medicines Agency (EMA) and FDA have developed common application forms for orphan drug designation to facilitate regulatory approval, and the creation of the European Committee for Orphan Medicinal Products has been essential to develop this collaboration and others between EMA and the World Health Organization (WHO).

Although the regulation has also been deemed as a success by promoting the development of drugs and EU biotech companies ( Horgan *et al*., 2020), 38% of market authorizations by 2010 were granted “under exceptional circumstances”, meaning that the applicant could not be expected to provide comprehensive evidence on the safety and efficacy given the rarity of the indication. Six percent of all approvals were conditional, indicating that further studies were needed in order to maintain the authorization ( Committee for Orphan Medicinal Products and the European Medicines *et al*., 2011).

The analysis by Giannuzzi and collaborators (2011) showed that between 2000 and 2015, significantly fewer ODDs were granted on average in the EU compared to the US, and the number of orphan drugs reaching the market was also significantly lower. In both regions, the field of oncology had the highest number of approvals, while a large portion of genetic rare diseases still face unmet therapeutic need ( Giannuzzi *et al*., 2017).

Regarding the type of applicant for ODDs, the analysis of Murakami and Narukawa (2016) shows similar patterns in the EU and US, with the top 100 pharmaceutical companies (defined by revenue ranking in 2013), holding 30.7% of all designations in the US (with the top 10 companies holding 15.4% of all designations) and 23.9% in the EU (with the top 10 companies holding 9.9% of all designations). Academic and other institutions held 4.7% and 6.2% of designations in the US and EU, respectively ( Murakami & Narukawa, 2016).

4.3. Orphan drug incentives in low- and middle-income countries

The discussion of impact of incentives for rare diseases in Low- and Middle-Income Countries originates with the definition of orphan drugs itself. As Herder says, “market forces can orphan diseases, either because they afflict those with purchasing power or because they affect so few in number”. With this idea, the author recalled in 2013 the consequences of the “calculated decision” to reduce “the scope of the problem” to those diseases that are rare in terms of low prevalence in the US ( Herder, 2013), but not those that are orphaned because they affect “developing nations” as Asbury highlighted ( Asbury, 1981, 1992), or “neglected diseases of the South” ( Herder, 2013). This conceptualization, combined with the market-oriented set of incentives derived in the neglect of diseases that were prevalent mostly in LMICs, is what has been denominated Neglected Diseases ( Cheung *et al*., 2004; Herder, 2013; Huyard, 2009; Trouiller *et al*., 2002). As an example of the market-oriented incentives, Sunyoto and collaborators (2018) use miltefosine (a drug used to treat neglected tropical diseases such as leishmaniasis) as a case-study of a drug that although it received orphan status, still faced problems of accessibility and availability in endemic countries ( Sunyoto *et al*., 2018), showing the limitations of the incentives contained in the ODA.

Some authors claim, however, that although these incentive packages do not target the development of drugs to treat Neglected Diseases, there may be some indirect benefits. As these diseases can be considered orphan in the US and Europe, the incentives could attract developers in this area ( Asbury, 1992; Dear *et al*., 2006). The literature offers examples of treatments that were assisted by the OOPD for malaria, tuberculosis and leprosy, and research candidates for Chagas disease, leishmaniasis, and yellow fever, as examples of how orphan drug regulation can support the development of therapies for neglected diseases ( Haffner *et al*., 2008; Haffner & Maher, 2006).

On the other hand, some authors highlight the unequal distribution of resources such as technology or knowledge as a root cause of the lack of R&D in these diseases, showing how most drug approvals did not correspond to the burden of disease in LMICs. This infers that the lack of stable demand makes Orphan Drug Act-like incentive packages non-ideal to boost R&D for neglected diseases of poverty ( Cheung *et al*., 2004; Trouiller *et al*., 2002).

When discussing the potential implementation of ODA-like policies in China, Liu and collaborators (2010) identify four key elements for a potential new legislation in the country: the definition of “rare diseases” in China, types of incentives for orphan drug R&D, price control, and healthcare practitioners' training. Concerning the definition, the authors comment on the data limitations to estimate the prevalence of some rare diseases in China, proposing the following definition for rare diseases:

“A life-threatening or seriously debilitating disease affecting fewer than 400,000 people (0.02–0.04 per cent of the population) could be regarded as a rare disease if: (1) no alternative treatments exist or the expected safety or pharmaceutical effect of new drugs might be significantly better than that of available drugs; (2) the cost of developing and distributing drugs to treat these rare and/or neglected diseases is unlikely to be recovered from the sales of the drug in Chinese territory; and (3) successful development can, based on theoretical analysis as in Japan, be expected.”

The authors propose a set of incentives that include a transparent national and local official registration system to monitor prevalence of diseases, fast-track approval for new orphan drugs when they have been approved in other countries, and tax exemptions and R&D push funds to stimulate new drug innovation in the country. Other mechanisms included collaboration with foreign regulatory authorities and academic institutions ( Liu *et al*., 2010).

Cheng & Xie (2017) commented on the potential development of a rare disease ecosystem in China that includes expedited review for rare disease drugs (although the authors mention that the pathway is not well defined), increase in diagnosis capacity, and the creation of coordination and information networks that include collaboration with patient groups to define the burden of rare diseases and raise awareness in the country ( Cheng & Xie, 2017). Additionally, Chan and collaborators (2020) capture in the supplemental materials that granting patents is the only policy directed towards orphan drug R&D in China ( Chan *et al*., 2020).

### Limitations

The research synthesis included only articles written in English, which can skew the results towards Anglophone countries and regions. In fact, the majority of the articles reviewed focused on the United States’ Orphan Drug Act, with only a few focusing on European and Japanese policies and regulations. The number of articles related to Low- and Middle-Income Countries (LMICs) was minimal and very little information was found regarding public policies directed to the development of new orphan drugs in these countries.

Another limitation is the minimal number of articles focused on the impact of orphan drug R&D policies on global access. This is likely linked to one of the exclusion criteria chosen for this synthesis, which was excluding publications solely focused on pricing and reimbursement of Orphan Drugs. Although it is impossible de-couple these downstream access issues from the incentives that facilitate R&D, the objective was to focus on those articles that studied specific innovation policies and their impact upstream and downstream. This might have skewed the selection of literature towards publications focused on High-Income Countries (HICs), excluding literature on access to orphan drugs in other regions.

## Conclusions

This paper offers a synthesis of the literature available in English on incentives for rare disease R&D. The literature shows how public policies in the US, followed by Japan, the EU, and others created a supportive environment for the development of innovative orphan drugs. These public policies cover the entire lifecycle of medicine development, from early-stage funding and regulatory support, to tax incentives and marketing exclusivity. Although these policies seem to have had a positive impact on orphan drug development, as judged by the increase of drugs treating rare diseases, there is substantial evidence of negative or spillover effects as well, such as the unaffordability of drugs or neglect of certain areas and diseases.

The literature reviewed in this synthesis also highlights the relationship between these incentives and the different actors in the system. Actors like patient groups have been essential in the development of some of the incentive programs, as well as funding and facilitating the R&D process in different ways. However, access to orphan drugs remains a problem for many of these same patients.

On the other hand, some authors point at these policies as a contributing factor of certain structural changes in the R&D system, with the emergence of actors like smaller biotechnology companies that focus on earlier stages of orphan drug development, and larger firms that focus more on later stages of development. With several orphan drugs becoming blockbusters, some authors suggest that these incentives created an “orphan drug business model” or “orphan drug strategy”, as an alternative to the traditional business model focused on mass production and sales of drugs.

This orphan drug business model seems to be stemming from or related to a certain misuse or gaming of the incentives. For instance, the accumulation of market exclusivity rights by obtaining multiple orphan drug designations for one drug has been linked to unaffordable prices for many orphan drugs. This has been facilitated through practices such as ‘salami slicing’ indications, subdividing diseases into smaller sub-diseases to obtain multiple designations, and the subsequent off-label use in more indications. In addition, while a lot of the innovative work in rare diseases is heavily focused on oncology, other rare diseases remain underserved, and issues related to clinical trial design and quality of regulatory dossiers have been raised. Finally, these policies have not been useful to address other areas such as neglected diseases that affect vulnerable populations mostly in LMICs.

In conclusion, this synthesis sheds light on the role of public policies driving pharmaceutical innovation in a previously neglected area. It also highlights the role of different actors taking part in rare disease R&D, as well as some of the negative consequences of these public policies. Finally, in an effort to look toward the future, this synthesis provides several of the proposals offered in the literature that can be used to overcome the existing negative consequences of the rare disease R&D status quo, and promote the goal of making drugs for rare diseases more affordable and accessible to all.

## Data Availability

No data are associated with this article.

## Footnotes

^1^
From Wellman-Labadie and Zhou (2010): “For the purposes of this study, biotechnology companies are generally defined as “emerging firms with limited cash reserves which develop novel, often first-in-class, large molecule-based drugs” as described by Malik”.

^2^
These articles reported COI as they were written by employees of pharmaceutical companies active in orphan drug development.
