# Supplementary material for: Pharmaceutical policy and innovation for rare diseases: A narrative review
Source: F1000Res. 2023 Nov 13;12:211. Originally published 2023 Feb 24. [Version 2] doi: 10.12688/f1000research.130809.2 (PMC11109548; doi:10.12688/f1000research.130809.2)
Supplement: Peer review history

# Reviewer report: Reviewer response for version 2

**DOI:** 10.5256/f1000research.158730.r272629

**Published:** 2024-05-24

**Authors:** Prof. Parveen Kumar Goyal

**Affiliations:** 1 Panipat Institute of Engineering and Technology (PIET), Haryana, India

Firstly, thank you for giving me a chance to review this report entitled “Pharmaceutical policy and innovation for rare diseases: A narrative review”. The authors have meticulously drafted the manuscript and efficiently tried to describe the pros and cons of ODA and other policies. I found the overall literature interesting, however, some below-mentioned minor issues, if addressed can make the manuscript more clear to the readers.

1. Authors have reviewed the literature points of referred authors effectively but have not stated their own points of view/ suggestions/ proposals about the designation of the orphan drug(s) and the pros and cons of associated policies.
2. The inclusion of brief text in the introduction section, about how this manuscript will be of interest to different readers such as policymakers, researchers, academicians, industry people, etc can make it a better point of concern.
3. There are several blind statements in the manuscript texting such as ‘increase in/ the number’, ‘decrease in/ the number’, ‘some authors said about the orphan drug’, etc without stating any referred numbers or range of figures, any name of the specific orphan drug(s), etc. It shall be of interest to the readers if authors can include some case studies and/ or illustrations to make the manuscript more specific.
4. The authors have limited their survey to PUBMED and included very few recent references. It would be appreciable if some more recent references could be added or updated as feasible.

Is the review written in accessible language?

Yes

Are all factual statements correct and adequately supported by citations?

Yes

Are the conclusions drawn appropriate in the context of the current research literature?

Yes

Is the topic of the review discussed comprehensively in the context of the current literature?

Yes

Reviewer Expertise:

Drug Development

I confirm that I have read this submission and believe that I have an appropriate level of expertise to confirm that it is of an acceptable scientific standard, however I have significant reservations, as outlined above.

# Reviewer report: Reviewer response for version 2

**DOI:** 10.5256/f1000research.158730.r272631

**Published:** 2024-05-21

**Authors:** Arianna Gentilini

**Affiliations:** 1 The London School of Economics and Political Science, London, England, UK

Thank you for the chance to review this update to the review. Overall, I found your literature review very interesting, timely, and well-written.

 However, there are a few issues you might want to consider, which I suggest below: 

  

- On a general note, hyperlinking is inconsistent throughout the document. Sometimes, you hyperlink the reference when in-text, while sometimes you only do it when within brackets.

 

 Section 1

- In this section, you might want to discuss some ongoing initiatives to address the lack of burden of disease data in the rare disease space for further context. For example, in 2023, the European Burden of Disease Network launched the Task Force on Rare Diseases to address this gap by researching the health impact of rare conditions in Europe ( see: [ref 1] )
- Here, you could discuss the concept of medical plausibility used by the EMA which, to some extent, limit the salami slicing practice ( see: [ref 2] )
- Finally, you could discuss the differential probability of success across disease types (rare vs non-rare) earlier in the introductory section as it seems relevant for statements you have later in the text (see [ref 3])

 

 Section 2

- You seem to contrapose the claims that the ODA affected positively the innovation rate in the rare disease space with the fact that many drugs are financed by the public sector, especially at initial R&D stages. While I agree with this, I am unclear about the link between the two concepts. As far as I am aware, we do not have evidence to suggest that publicly-funded research groups/companies were immune to incentives by the ODA? Rather, I would emphasise the fact that there are many confounding factors, such as improvements in basic science (see the first DNA sequence happening around the same time) and in genomics, which are likely to have affected rare diseases, which are mostly genetic in nature, disproportionately.

Section 2.2

- It would be useful to add reference to the following statement "First, the market exclusivity clause has been portrayed as the major contributor to the success of the ODA, but also as a major driver of unaffordable prices, as the monopolies allow companies to set high prices without competition."
- You discuss EU-related issues in this section, which should focus solely on the negative consequences of the ODA. I would suggest moving this elsewhere, possibly in the next section.
- You frame your review as innovation in the rare disease space, but you specifically look at legal frameworks and do not look at - for example - policies beyond orphan regulations such as R&D investments and pricing and reimbursement frameworks. I wonder if the title is appropriate?

 

 Section 2.3

- It does not look like the ones you suggest can be defined solely as changes to the ODA specifically, but broader change to the incentive system in the US, e.g. repurposing. I suggest you change the title of the section or move the content elsewhere in the paper.
- You could justify better the primary relevance of the US regulation, considering the current set-up of the pharmaceutical system (i.e., the US making up the majority of sales etc.).

 

 Section 3

- In this section, you might want to reference Gentilini & Miraldo 2023 ( see [ref 4] ), which specifically looks at the role and impact of patient groups in innovation in the rare disease R&D space.
- Your last sentence discussing the risk of conflicts of interest between patient groups focusing on rare disease and industry is currently missing a reference. In this context, Gentilini & Parvanova 2023 ( see [ref 5] ) might be relevant, as it is the only paper discussing the share of industry funding directed to patient groups focusing on rare diseases specifically. 

Section 4.2

- When discussing European incentives, you could mention the changes suggested as part of the revision to the orphan regulation (see inception impact assessment here: [ ref 6 ]), which proposes market exclusivities of different lengths depending on the type of development (innovative products; re-purposed products; second/multiple indications). 
- While you hint at it in your second bullet point, you could explicitly say that the profitability clause has never been implemented (i.e., de facto orphan designation can only be granted based on the prevalence criterion).
- Please provide a reference for the following statement: "On the regulatory side, the Regulation provides protocol assistance and fee waivers (100% waiver for protocol assistance and 50% for all regulatory fees), which is highlighted as a strong incentive for small- and medium-sized enterprises. Protocol assistance can relate to the support for regulatory requirements such as product quality, preclinical studies, clinical trial design, etc."
- You mention 29 EU member states, but there are only 27? If you are including the UK and Switzerland please add them separately as Norway and Iceland.

 

 Section 4.3

- In the LMICs section, you could explicitly refer to the willingness to pay issues between different regions, which inevitably translates into different degrees of attractiveness to for-profit companies.
- I think that competition in rare diseases would deserve mention. For example, many orphan drugs will be coming off-patent in the next years, which is expected to increase competition and lower prices. However, there is evidence that competition in rare diseases is low and mostly occurs in conditions that are more prevalent or have a higher willingness-to-pay, such as cancer or cystic fibrosis (see [ ref 7 ] ). The question then is, how will these drugs be affordable in LMICs? Joint procurement? Price discrimination based on GDP?
- It might be worth mentioning that both China and India have made progress in their rare disease policy. For example, China set up its first rare diseases registry in 2020 (see [ ref 8 ]), while India published its National Rare Diseases Policy India in 2021 (see [ ref 9 ])

 

 Limitations

- You might also mention as a limitation the fact that you did not consider governmental documents and legislations but only peer-reviewed literature, which might have overlooked some issues and initiatives.

Is the review written in accessible language?

Yes

Are all factual statements correct and adequately supported by citations?

Partly

Are the conclusions drawn appropriate in the context of the current research literature?

Yes

Is the topic of the review discussed comprehensively in the context of the current literature?

Yes

Reviewer Expertise:

I am a researcher focusing on rare disease policy, with a focus on high-income countries.

I confirm that I have read this submission and believe that I have an appropriate level of expertise to confirm that it is of an acceptable scientific standard.

## References

[1] Reference source

[2] Reference source

[3] : Estimation of clinical trial success rates and related parameters. *Biostatistics* .2019;20(2) : 10.1093/biostatistics/kxx069 273-286 10.1093/biostatistics/kxx069 29394327 PMC6409418

[4] Reference source

[5] Reference source

[6] Reference source

[7] : Generic Competition for Drugs Treating Rare Diseases. *J Law Med Ethics* .2020;48(4) : 10.1177/1073110520979391 789-795 10.1177/1073110520979391 33404340

[8] Reference source

[9] Reference source

# Reviewer report: Reviewer response for version 2

**DOI:** 10.5256/f1000research.158730.r272633

**Published:** 2024-05-10

**Authors:** Joel R. Lexchin

**Affiliations:** 1 School of Health Policy & Management, Faculty of Health, York University, Toronto, Canada; 2 University of Toronto, Toronto, Canada

This is an excellent report summarizing the literature about the positives and negatives primarily of the US Orphan Drug Act but also mentioning the legislation in the EU and in Japan. With orphan drugs making up an increasing percent of new drug approvals this article is extremely important to help us understand the reasons behind the evolution of drug R&D. I’m also happy that the article points out the difference between industry’s enthusiasm for bringing drugs to market for orphan diseases and industry’s neglect of drugs for neglected diseases.

 There are a few relatively minor issues that I think that the authors could address:

1. Why was the search confined to PubMed and additional databases not used?
2. Did the authors identify topics related to orphan drugs that had not been evaluated in the literature? If so, they should mention what additional topics need to be researched.
3. Results, third paragraph: Is there any data about what percent of drugs were marketed for orphan diseases before the Kefauver-Harris amendments?
4. Last paragraph in Section 2: The point about expanding market exclusivity to patented products was already made on the previous page.
5. Section 2.1, second paragraph: Did any of the studies assess the additional therapeutic value of drugs developed through the ODA compared to existing therapies?
6. Section 4.1: Express the amount in Japanese yen in other currencies, e.g., American dollars, Euros, British pounds.

Is the review written in accessible language?

Yes

Are all factual statements correct and adequately supported by citations?

Yes

Are the conclusions drawn appropriate in the context of the current research literature?

Yes

Is the topic of the review discussed comprehensively in the context of the current literature?

Yes

Reviewer Expertise:

Pharmaceutical policy

I confirm that I have read this submission and believe that I have an appropriate level of expertise to confirm that it is of an acceptable scientific standard, however I have significant reservations, as outlined above.

# Reviewer report: Reviewer response for version 1

**DOI:** 10.5256/f1000research.143589.r193380

**Published:** 2023-09-06

**Authors:** Daniel Michaeli

**Affiliations:** 1 Department of Personalized Oncology, University Hospital Mannheim, Heidelberg University, Baden-Wuerttemberg, Mannheim, Germany

Thank you for giving me the opportunity to review the manuscript entitled "Pharmaceutical policy and innovation for rare diseases: A narrative review". This is an important article. I suggest to accept it for indexing after minor revisions. There are certain aspects missing in the authors' review of the ODA;

 The authors correctly mention that companies have begun to "slice" common diseases into multiple narrow orphan subgroups with the help of precision medicine. This point should be elaborated on with the help of the following articles. Furthermore, the authors should present policy solutions for this issue (as presented by the following articles), and discuss the differences between FDA vs. EMA.

1. Michaeli *et al.* (2023 ^1^).
2. Kesselheim *et al.* (2017 ^2^).
3. Vokinger *et al.* (2019 ^3^).
4. Michaeli D. and Michaeli T. (2023 ^4^).

The authors should mention two recent studies that evaluated the impact of the FDA orphan designation on pharmaceutical company valuation and returns that investors and entrepreneurs can expect from investing companies developing orphan drugs:

1. Michaeli *et al.* (2022 ^5^).
2. Michaeli *et al.* (2022 ^6^).

The authors should be specific about their results and implications in the abstract (don't just state what you are going to do, but provide us, readers, with key insights) and policy solutions of the highlighted very important challenges.

 The authors have not yet mentioned the issue of partial orphan drugs, e.g. drugs that are approved to treat common and rare diseases. Please read more on this topic in the following references and cite them appropriately:

1. Michaeli D. and Michaeli T. (2023 ^7^).
2. Chua *et al.* (2021 ^8^).
3. Gunter *et al.* (2020 ^9^).

Is the review written in accessible language?

Yes

Are all factual statements correct and adequately supported by citations?

Partly

Are the conclusions drawn appropriate in the context of the current research literature?

Yes

Is the topic of the review discussed comprehensively in the context of the current literature?

Partly

Reviewer Expertise:

oncology, health policy, drug development, clinical trials, drug pricing

I confirm that I have read this submission and believe that I have an appropriate level of expertise to confirm that it is of an acceptable scientific standard, however I have significant reservations, as outlined above.

## References

[1] : FDA approval, clinical trial evidence, efficacy, epidemiology, and price for non-orphan and ultra-rare, rare, and common orphan cancer drug indications: cross sectional analysis. *BMJ* .2023;381: 10.1136/bmj-2022-073242 e073242 10.1136/bmj-2022-073242 37160306 PMC10167557

[2] : Biomarker-Defined Subsets of Common Diseases: Policy and Economic Implications of Orphan Drug Act Coverage. *PLoS Med* .2017;14(1) : 10.1371/journal.pmed.1002190 e1002190 10.1371/journal.pmed.1002190 28045970 PMC5207392

[3] : Application of orphan drug designation to cancer treatments (2008-2017): a comprehensive and comparative analysis of the USA and EU. *BMJ Open* .2019;9(10) : 10.1136/bmjopen-2018-028634 e028634 10.1136/bmjopen-2018-028634 31601584 PMC6797305

[4] : Spending on Orphan Cancer Drugs for Ultra-Rare, Rare, and Common Diseases. *SSRN Electronic Journal* .2023; 10.2139/ssrn.4421181 10.2139/ssrn.4421181

[5] : Valuation and Returns of Drug Development Companies: Lessons for Bioentrepreneurs and Investors. *Ther Innov Regul Sci* .2022;56(2) : 10.1007/s43441-021-00364-y 313-322 10.1007/s43441-021-00364-y 35018622 PMC8854317

[6] : Value drivers of development stage biopharma companies. *Eur J Health Econ* .2022;23(8) : 10.1007/s10198-021-01427-5 1287-1296 10.1007/s10198-021-01427-5 35038054 PMC9550717

[7] : Partial Orphan Cancer Drugs: FDA Approval, Clinical Benefit, Trials, Epidemiology, Price, Beneficiaries, and Spending. *SSRN Electronic Journal* .2023; 10.2139/ssrn.4538921 10.2139/ssrn.4538921 38244983

[8] : Spending For Orphan Indications Among Top-Selling Orphan Drugs Approved To Treat Common Diseases. *Health Aff (Millwood)* .2021;40(3) : 10.1377/hlthaff.2020.01442 453-460 10.1377/hlthaff.2020.01442 33646878 PMC8011952

[9] : Medicaid Spending on Drugs Marketed Without US Food and Drug Administration Approval in 2020. *JAMA Intern Med* .2022;182(3) : 10.1001/jamainternmed.2021.7614 342-345 10.1001/jamainternmed.2021.7614 35040875 PMC8767485

# Response

**Published:** 2023-11-07

**Authors:** Adrián Alonso Ruiz

**Affiliations:** Global Health Centre, Graduate Institute of International and Development Studies Geneva, Switzerland

We thank the feedback received from the reviewer. His valuable contributions have improved the manuscript substantially, highlighting the importance of “partial Orphan Drugs” and the contribution of Orphan Drug incentives in companies’ valuation. Afer reviewing the literature recommended by the reviewer, we have incorporated these relevant features of the Orphan Drug business model, as well as some of the solutions included.

# Reviewer report: Reviewer response for version 1

**DOI:** 10.5256/f1000research.143589.r164970

**Published:** 2023-03-24

**Authors:** Claudia Vaca González

**Affiliations:** 1 Department of Pharmacy, Faculty of Sciences, National University of Colombia, Bogotá, Colombia

This literature review is handy on how the market rationale captured the orphan drug R&D policies and became them in a business strategy to take advantage of both public financing and extending market exclusivity. The US, European, and Japanese orphan drug R&D policies are well described in the paper, as well as its affordability and sustainability failures. Also, the article provides evidence about the undesirable outcomes of the current R&D model and some recommendations to fix it.

 On the other hand, as the authors explain, given the few articles they find on Low- and Middle-Income Countries (LMICs), it is hard to extrapolate the findings. In the same line, and given the definition of orphan drugs is far from neglected diseases, the authors could consider removing the phrase "these policies have not helped address other areas, such as neglected diseases that affect vulnerable populations, mostly in LMICs." 

 Finally, because of few papers found on R&D policies in LMIC, the authors could suggest another research question around the negative impact of the orphan drug business strategy on the drug regulatory systems and access to orphan drugs in these countries, carrying direct interviews, grey reviews, etc.

Is the review written in accessible language?

Yes

Are all factual statements correct and adequately supported by citations?

Yes

Are the conclusions drawn appropriate in the context of the current research literature?

Yes

Is the topic of the review discussed comprehensively in the context of the current literature?

Yes

Reviewer Expertise:

Pharmacoepidemiology and Public Health

I confirm that I have read this submission and believe that I have an appropriate level of expertise to confirm that it is of an acceptable scientific standard.

# Response

**Published:** 2023-11-07

**Authors:** Adrián Alonso Ruiz

**Affiliations:** Global Health Centre, Graduate Institute of International and Development Studies Geneva, Switzerland

We appreciate the feedback received from the reviewer, and thank her for her decision to approve the publication of the manuscript. We have taken into consideration her recommendation to nuance the language regarding the impact of policies for rare disease research and development; in the incentivizing the development of new treatments for neglected diseases in LMICs. In that sense, we have complemented the text to clarify that despite qualifying as rare diseases in most of the countries where these incentives are implemented, many diseases prevalent in LMICs remain unattended.
